# Nocardial Infection in the Early Period after Kidney Transplantation

**DOI:** 10.1155/2022/2252825

**Published:** 2022-08-12

**Authors:** Sudeendra Gupta, Ammar Abdulbaki, Sandra El Hajj, Ahmad Nusair, Mohamad Mooty, Nizar Attallah

**Affiliations:** ^1^Medical Sub-specialties Institute, Department of Nephrology, Cleveland Clinic, Abu Dhabi, UAE; ^2^Cleveland Clinic Lerner College of Medicine of Case Western University, Cleveland, OH, USA; ^3^Department of Pharmacy, Cleveland Clinic, Abu Dhabi, UAE; ^4^Department of Infectious Diseases, Cleveland Clinic, Abu Dhabi, UAE

## Abstract

Patients with solid organ transplant have weaker immune system and can develop opportunistic infections. Prophylactic antimicrobials can help lower that risk but do not prevent it completely. High index of suspicion increases the chance of diagnosing rare opportunistic infections in immunocompromised patients and helps early and effective treatment. We present a unique case of a patient who developed pneumonia from *Nocardia* early after kidney transplant despite being on trimethoprim-sulfamethoxazole (TMP-SMX) prophylaxis. He was diagnosed and treated early which helped improving his outcome. We discuss incidence, risk factors, and treatment of nocardiosis post kidney transplant.

## 1. Introduction

Solid organ transplant offers patients better survival and quality of life. Yet, patients with solid organ transplantation (SOT) are susceptible for opportunistic infections as they are on chronic immunosuppressive medications. Use of antimicrobial prophylaxis in the initial 6–12 months mitigates the risk of some opportunistic infections. Despite that, some patients can develop opportunistic infections. We report here a case of nocardiosis in the early transplant period and the therapeutic challenges we faced with management.

### 1.1. Case Report

A 64-year-old male with past medical history of end-stage renal disease due to type II diabetes mellitus underwent hemodialysis for two years prior to receiving deceased donor kidney transplantation on 13^th^ May, 2019. He was an ex-smoker and quit smoking 5 months before his transplant surgery. He received basiliximab 20 mg (on day 0 and day 4) and intravenous methylprednisolone as induction immunosuppression followed by triple drug maintenance immunosuppression regimen of tacrolimus (with target trough level of 6–8 ng/mL), mycophenolic acid 540 mg twice daily, and steroid taper per our protocol. His immediate post-transplant course was uneventful, and his serum creatinine nadir reached 1.1 mg/dl (97.4 *μ*mol/L). He was also on antimicrobial prophylaxis with trimethoprim-sulfamethoxazole, oral nystatin, and valganciclovir as per protocol.

**Figure 1:**
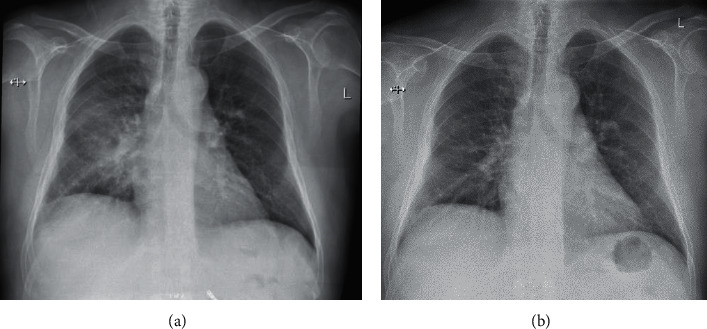
Chest X-ray showing right lower zone non-homogenous opacity with air bronchogram (a). Then, there was remarkable improvement 6 months later (b).

**Figure 2:**
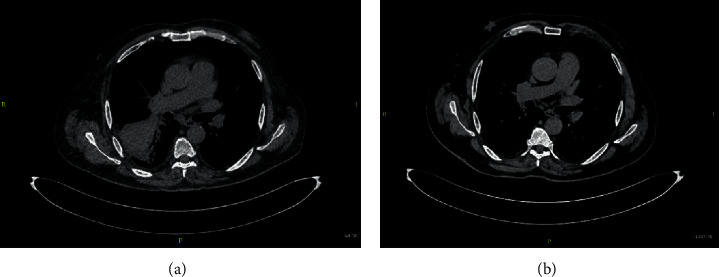
CT image showing pneumonic consolidation in the right lower zone (a) which improved remarkably with treatment 12 months later (b).

On 28^th^ June, 2019, he presented to the emergency department with fever of 38.4°C, shortness of breath, cough with mucopurulent expectoration, and pleuritic chest pain for three days. He did not have any nausea or vomiting, nasal congestion, or sore throat and denied any sick contact. Clinical examination revealed ill looking, febrile patient with blood pressure of 110/50 mmHg and heart rate of 94/min. Chest examination revealed crepitations in the right lower field. Examination of other systems was unremarkable. His chest X-ray (CXR) revealed ill-defined consolidation with air bronchogram suspicious for right lower lobe pneumonia (Figure 1(a)). His laboratory investigations showed hemoglobin of 9.9 g/dl, total white blood counts of 9800/mm^3^, platelet counts of 123000/mm^3^, serum creatinine of 1.59 mg/dl (140.9 *μ*mol/L), blood urea nitrogen (BUN) of 73 mg/dl, and tacrolimus trough level of 10.1 ng/mL. Urinalysis and liver function tests were unremarkable. The patient was treated with broad-spectrum antibiotics (piperacillin-tazobactam, azithromycin, and vancomycin) for pneumonia in an immunocompromised patient. Antibiotics were escalated after two days to meropenem, levofloxacin, and vancomycin due to persistent fever. CT chest revealed consolidation in the right lower lobe (Figure 2(a)). No endobronchial masses were identified in the bronchi subtending the consolidated pulmonary segments. Immunosuppressive regimen was also modified due to presence of active infection, where tacrolimus target was reduced to 5–7 ng/mL, and mycophenolic acid dose was lowered initially to 360 mg twice daily for 3 days, then it was held completely since the patient was not getting better. Since he did not improve quickly, a more expanded sputum culture was done and that grew after three days *Nocardia* species. Meropenem was continued and trimethoprim-sulfamethoxazole (TMP-SMX) was modified to therapeutic dosages. Two days later, TMP-SMX was switched to minocycline due to recurrent hyperkalemia. CT brain was done to rule out any brain abscesses from *Nocardia* which showed a small old lacunar infarct of the right basal ganglia. Patient improved and was sent home in a stable condition. He was discharged on oral minocycline 100 mg twice daily. Of note, *Nocardia* isolate sensitivity came back couple of weeks later, and it was sensitive to TMP-SMX and linezolid. There was intermediate sensitivity to minocycline. Clinical decision was made to continue minocycline for 12 months given significant clinical improvement and lack of alternatives since the patient developed severe hyperkalemia on TPM-SMX. Linezolid was not used for fear of long-term myelosuppressive effects. He was followed regularly in Renal Transplant Clinic and Infectious Disease Clinic.

Imaging and clinical status were monitored closely. CXR at 6 months showed significant improvement (Figure 1(b)), and CT of the chest repeated at 12 months showed complete resolution (Figure 2(b)).

## 2. Discussion

Nocardiosis is an opportunistic infection caused by aerobic *Actinomycete*, a Gram-positive filamentous bacterium. People with exposure to soil or decaying vegetation are at risk of catching the infection. With intact cell-mediated immunity, it is rare to develop this infection. Patients on immunosuppressive medications especially SOT recipients are at risk of developing nocardiosis. Other risk groups are patients with stem cell transplant, HIV, and chronic pulmonary diseases.

The incidence of nocardiosis in SOT is found to be 0.04 to 3.5% [1]. In recent times with better detection and identification procedures, it has been felt that nocardial infections have been more than what it used to be 2 decades ago. The population of immunosuppressed patients like post SOT is also expanding which is likely also another reason for increasing incidence [2].

Lung transplanted patients have a higher risk of developing nocardiosis when compared to other solid organ transplants [1].

A definitive diagnosis of nocardiosis requires the isolation and identification of the organism from a clinical specimen [3]. Establishing a diagnosis of nocardiosis is problematic since an invasive procedure is often required to obtain an adequate specimen and recovery of *Nocardia* in the laboratory is difficult because of its slow growth. In routine aerobic cultures, nocardia usually requires 5-21 days for growth. Polymerase chain reaction provides more accurate and rapid results for the diagnosis of nocardiosis than the conventional methods, but it is not available in many clinical laboratories. Precise speciation and susceptibility testing of clinical isolates is important because resistance patterns vary by species. In the proper clinical setting, a presumptive diagnosis of nocardiosis can be made if partially acid-fast filamentous branching rods are visualized in clinical specimens [3].

Two case control studies with different/sequential timelines have identified various risk factors for nocardiosis in SOT recipients. They included recipient older age at diagnosis, older donor age, length of stay in ICU after transplantation, diabetes, history of blood stream infection between the period of transplantation and nocardiosis, acute rejection in the 6-month period before the diagnosis of nocardiosis, high trough concentrations of calcineurin inhibitors within the previous month of diagnosis of nocardiosis, use of tacrolimus, use of high dose of corticosteroids in the 6-month period preceding the diagnosis of nocardiosis, use of plasma exchange or depleting antibodies for induction, CMV infection, and lower lymphocyte counts in the preceding months [1, 4, 5].

Typically, nocardial infections happen more than 6 months after solid organ transplant during the period of routine use of antimicrobial prophylaxis for *Pneumocystis* infections with co-trimoxazole [6]. Use of low-dose co-trimoxazole chemoprophylaxis usually given for prevention of *Pneumocystis jiroveci* pneumonia post-transplant may not be protective for nocardiosis in all patients.

In our patient, the infection happened earlier. This could mean that the patient was colonized with *Nocardia* species before the transplant, and they got reactivated or that he got exposed directly to *Nocardia* soon after transplantation.

In earlier series, most common *Nocardia* species was *N. asteroides* (∼90%) followed by *N. brasiliensis*, *N. caviae*, and others [7]. With advancements in molecular methods for species identification, many new species have been identified. There has been some variability in the incidences of different species in SOT as per some series mainly due to variability in detection methods [2].

Pulmonary involvement is the most common finding in nocardiosis followed by skin and brain [7]. Brain involvement can be insidious, and, in many cases, clinical neurological examination can be normal. Hence, a neuroimaging (CT or MRI) is justified in a case of solid organ transplantation and invasive nocardiosis even in the absence of symptoms or signs related to the nervous system [1].

Our patient was an elderly male who presented with fever and chest symptoms suggestive of pneumonia. On microbiological investigations of sputum, nocardiosis was found.

His CT brain was done to rule out brain abscess, and fortunately it was unremarkable.

He was treated with broad-spectrum antibiotics initially on admission, and once sputum culture revealed *Nocardia*, definitive therapy with co-trimoxazole was started. In view of moderate to severe hyperkalemia, co-trimoxazole was switched to minocycline for 12 months.

At the end of 12 months, CT chest showed complete resolution of pneumonia secondary to nocardiosis.

Our patient had pulmonary manifestations of nocardiosis in a relatively early post-transplant period of 6 weeks despite being on co-trimoxazole prophylaxis. The purpose of presenting this case is to increase awareness about opportunistic infections like *Nocardia* which can present very early due to various reasons discussed despite being on chemoprophylaxis and should be considered in differential diagnosis of patients presenting with fever in the post-transplant setting. Risk factors in our patient included older age, solid organ transplantation, and diabetes.

Early recognition is very important to initiate early treatment. Our patient had hyperkalemia which limited further continuation of co-trimoxazole but fortunately responded to minocycline.

## 3. Conclusions

High index of suspicion for nocardiosis should be maintained in transplant recipients presenting with fever and persistent pulmonary symptoms even as early as one month post-transplant. Early detection and treatment can lead to favourable outcomes. Alternatives to TMP-SMX can be of help in patients who are intolerant to TMP-SMX.

## Data Availability

No data were used to support this study.
